# Protein Profiles Reveal Diverse Responsive Signaling Pathways in Kernels of Two Maize Inbred Lines with Contrasting Drought Sensitivity

**DOI:** 10.3390/ijms151018892

**Published:** 2014-10-20

**Authors:** Liming Yang, Tingbo Jiang, Jake C. Fountain, Brian T. Scully, Robert D. Lee, Robert C. Kemerait, Sixue Chen, Baozhu Guo

**Affiliations:** 1 USDA-ARS, Crop Protection and Management Research Unit, Tifton, GA 31793, USA; E-Mails: yanglm@uga.edu (L.Y.);brian.scully@ars.usda.gov (B.T.S.); 2 Department of Plant Pathology, University of Georgia, Tifton, GA 31793, USA; E-Mails: jfount1@uga.edu (J.C.F.); kemerait@uga.edu (R.C.K.); 3 School of Life Sciences, Huaiyin Normal University, Huaian 223300, China; 4 State Key Laboratory of Tree Genetics and Breeding, Northeast Forestry University, Harbin 150040, China; E-Mail: tbjiang.usa@gmail.com; 5 Department of Crop and Soil Sciences, University of Georgia, Tifton, GA 31793, USA; E-Mail: deweylee@uga.edu; 6 Department of Biology, Genetics Institute, and Plant Molecular & Cellular Biology Program, University of Florida, Gainesville, FL 32611, USA; E-Mail: schen@ufl.edu

**Keywords:** maize kernel, differentially expressed proteins, drought stress, iTRAQ-based proteomics, signaling pathways

## Abstract

Drought stress is a major factor that contributes to disease susceptibility and yield loss in agricultural crops. To identify drought responsive proteins and explore metabolic pathways involved in maize tolerance to drought stress, two maize lines (B73 and Lo964) with contrasting drought sensitivity were examined. The treatments of drought and well water were applied at 14 days after pollination (DAP), and protein profiles were investigated in developing kernels (35 DAP) using iTRAQ (isobaric tags for relative and absolute quantitation). Proteomic analysis showed that 70 and 36 proteins were significantly altered in their expression under drought treatments in B73 and Lo964, respectively. The numbers and levels of differentially expressed proteins were generally higher in the sensitive genotype, B73, implying an increased sensitivity to drought given the function of the observed differentially expressed proteins, such as redox homeostasis, cell rescue/defense, hormone regulation and protein biosynthesis and degradation. Lo964 possessed a more stable status with fewer differentially expressed proteins. However, B73 seems to rapidly initiate signaling pathways in response to drought through adjusting diverse defense pathways. These changes in protein expression allow for the production of a drought stress-responsive network in maize kernels.

## 1. Introduction

Field crops endure various environmental stresses throughout their life cycle, which affect their growth and development and, consequently, hamper crop yield and quality [1]. Among these environmental factors, drought is considered the single most devastating stress, decreasing crop productivity more than any other abiotic factor [2]. Moreover, global warming may be worsening this situation in most agricultural regions [2].

As a cereal crop with a large annual global production, maize yield and quality are crucial agronomic traits, which are continuously constrained and destabilized due to drought [3]. Though affected throughout all growth stages, maize is most sensitive to drought stress during pollination and grain-filling [3]. In the Southern U.S., drought, along with continuously high temperatures during the maize reproductive stage, can not only contribute to poor kernel development, but can also weaken the defense capabilities of maize against pathogen attacks. For example, drought stress can exacerbate the possibility and severity of *Aspergillus flavus* infection and subsequent aflatoxin contamination in maize kernels [4,5,6,7]. Interestingly, as well, aflatoxin-resistant lines tend to also be drought tolerant [5,8,9].

An effective strategy to alleviate drought-related injury is to improve maize drought tolerance through developing novel lines through traditional breeding. However, the quantitative/multigenic nature of drought tolerance or sensitivity complicates efforts in maize breeding programs [10,11]. Although maize lines tolerant to drought have been identified [12,13], the introgression of drought tolerance from these lines into commercial cultivars is slow due to the lack of selective markers.

It has been demonstrated that maize resistance to drought stress is accomplished through the enhancing or inhibitory functions of various drought-responsive proteins, which regulate stress-responsive metabolic processes and pathways. Increased numbers and levels of protective and stress-related proteins in maize seedlings have been detected in tolerant lines in comparison to sensitive lines under drought stress, such as dehydrins, heat shock proteins, hydrolases, xylanase inhibitor and pathogenesis-related protein 10 (PR-10), which has similarity in sequence to the receptor families of the pyrabactin resistance 1 (PYR1)/PYR1-like (PYL)/regulatory components of ABA receptors (RCAR) that are involved in ABA signaling during plant response to abiotic stress [14].

In previous studies, Luo *et al.* [15] analyzed gene expression profiles across a temporal sampling interval in developing maize kernels under drought conditions and found that most genes responding to drought are expressed 35–40 days after pollination (DAP), which corresponds to an observed distinct phase in kernel development related to disease defense from 35–45 DAP. Jiang *et al.* [16] reported genotype-based expression pattern differences of PR and stress-related genes in response to drought stress in kernels of six inbred lines with different resistance to aflatoxin contamination, including the resistant inbred lines, A638, Lo964, Mp313E and Tex6, and susceptible inbred lines B73, Lo1016 and Mo17. Among these lines, Lo964 and B73 possess differential sensitivities and responses to drought stress with Lo964 being drought tolerant line [17,18]. In this study, therefore, a moderate degree of drought stress with 50% reduction in irrigation water was applied in order to examine the reaction of different maize lines to the applied stress through different signaling pathways. Identified proteins with a correlation to drought responses can be used as potential markers for screening maize lines for drought tolerance in breeding programs through marker-assisted selection (MAS) as the long-term goal.

## 2. Results and Discussion

### 2.1. Maize Kernel Proteome Patterns in Response to Drought Stress Treatments

Comparative proteomic analysis was used to investigate the changes of protein profiles in kernels of B73 and Lo964 under drought conditions. A total of 78 proteins were found to have significant expression changes (*p* ≤ 0.05); among them, 70 proteins from B73 and 36 proteins from Lo964 showed significant changes in field drought-treated samples compared to well-watered samples (Table 1; the detailed abundant changes of each protein mentioned above in response to drought are listed in Supplementary Table S2). This is an interesting observation, considering that B73 is a drought-sensitive line, while Lo964 can better tolerate drought-stress conditions [16].

**Table 1:** Differentially expressed proteins in maize kernels under drought stress treatment.

GI ^a^	Protein Name ^b^		Coverage (%) ^c^ /Peptide Fragments ^d^	Fold Change (B73) ^e^		Fold Change (Lo964) ^e^
Cell rescue/defense						
219363419	Dehydrin		23.0/6	1.64 ± 0.09		/
295856	RAB-17		37.5/10	1.36 ± 0.09		/
226491145	Late embryogenesis abundant protein Lea14-A		74.3/17	1.46 ± 0.15		/
195659191	Embryonic abundant protein 1		84.6/25	1.47 ± 0.15		−1.48 ± 0.08
226499304	Pathogenesis-related protein 10		11.2/2	1.27 ± 0.08		−1.30 ± 0.03
226531123	Glycine-rich protein 2b		44.2/3	1.61 ± 0.20		1.42 ± 0.09
226492587	Stress-inducible membrane pore protein		63.2/15	1.32 ± 0.07		/
226496775	Xylanase inhibitor protein 1		24.5/3	1.63 ± 0.04		−1.31 ± 0.02
293332305	Serpin-ZXA-like		13.6/5	1.30 ± 0.05		/
293335211	Heavy-metal-associated Domain-containing protein		11.9/2	1.54 ± 0.52		/
473187	Protein kinase C inhibitor		24.2/2	−1.46 ± 0.04		/
75994217	Hageman factor inhibitor		18.7/2	−1.45 ± 0.03		/
Redox homeostasis						
257333334	Glutathione transferase30		21.2/8	1.40 ± 0.14	−1.42 ± 0.02	
48374955	Glutathione peroxidase		44.0/8	1.33 ± 0.08	/	
226494622	Glutathione *S*-transferase,-like protein		19.8/3	1.44 ± 0.06	/	
257728955	Grx_C2.2—glutaredoxin subgroup I		69.9/8	1.26 ± 0.10	1.34 ± 0.09	
87133468	1-Cys peroxiredoxin antioxidant		76.4/57	1.32 ± 0.08	/	
195652835	Thioredoxin H-type		61.5/9	1.33 ± 0.09	/	
7548002	Mn-superoxide dismutase		47.6/18	1.59 ± 0.18	/	
6018746	Superoxide dismutase-4A		46.7/6	1.28 ± 0.06	/	
238008410	NADH-ubiquinone oxidoreductase subunit B17.2		18.2/2	1.53 ± 0.01	/	
195655511	NAD dependent epimerase/dehydratase		24.3/2	1.64 ± 0.05	1.35 ± 0.07	
Hormone response						
226497210	ABA-responsive protein		23.5/5	1.46 ± 0.04	/	
226498678	Ethylene-responsive protein		28.7/6	1.50 ± 0.19	/	
226508662	Ethylene-responsive protein		27.2/7	1.49 ± 0.15	/	
301069326	Auxin response factor 23		9.56/1	1.52 ± 0.19	1.35 ± 0.09	
224028639	Jasmonate-induced protein		26.8/2	−1.54 ± 0.008	−1.31 ± 0.17	
Signal transduction						
229611800	GTP-binding nuclear protein Ran-A1		40.1/11	−1.35 ± 0.04	−1.20 ± 0.01	
226528736	ATP/GTP binding protein		16.1/4	1.34 ± 0.08	/	
255037841	Histidine kinase		7.52/2	−1.51 ± 0.02	1.28 ± 0.11	
226493048	Nucleoside diphosphate kinase 4		23.5/4	1.35 ± 0.09	/	
254256262	Pyruvate orthophosphate dikinase 1		23.0/12	−2.11 ± 0.04	−1.43 ± 0.05	
Storage proteins						
228310	Globulin 2		77.6/156	/	−1.51 ± 0.12	
224030527	Globulin 1		70.5/205	1.41 ± 0.10	/	
195658011	Globulin-1 *S* allele precursor		77.6/152	1.63 ± 0.22	/	
226500532	Seed maturation protein PM41		18.0/9	1.42 ± 0.08	/	
22284	Vicilin-like embryo storage protein		69.8/180	/	1.46 ± 0.001	
330732090	γ-zein		2.27/1	−1.67 ± 0.01	−1.13 ± 0.006	
Protein biosynthesis						
9931636	Ribosomal protein s6 RPS6-2		23.1/5	1.65 ± 0.26	/	
257667240	40S ribosomal protein S18		54.6/7	1.52 ± 0.09	/	
6226702	40S ribosomal protein S8		33.5/7	1.81 ± 0.10	/	
226502084	60S ribosomal protein L7a		19.8/5	1.50 ± 0.14	/	
258598734	QM-like protein		23.7/4	1.36 ± 0.08	/	
Protein folding and assembly						
7546186		Heat shock protein HSP82	23.1/9	−1.65 ± 0.03		/
293331695		HSP protein	23.3/10	/		−1.51 ± 0.04
54299342		Mitochondrial small heat shock protein 22	44.0/7	/		−1.39 ± 0.02
293335765		TCP-1/cpn60 chaperonin family protein	8.94/2	/		−1.34 ± 0.09
453670		Heat shock protein 26	57.5/9	/		−1.44 ± 0.07
257745378		Peptidyl-prolyl cis-trans isomerase Family protein	19.5/3	/		1.29 ± 0.02
226495869		Histone deacetylase 6	6.49/2	1.45 ± 0.13		−1.91 ± 0.09
59861271		Protein disulfide isomerase	6.56/2	1.90 ± 0.29		/
Protein degradation						
224029787		Proteasome subunit α type	49.2/8	1.40 ± 0.12		−1.26 ± 0.04
226531007		Proteasome subunit α type 1	22.3/7	1.32 ± 0.08		−1.17 ± 0.02
Carbohydrate metabolism						
22488		Sucrose synthase	22.8/10	−1.73 ± 0.03		−1.42 ± 0.06
3342802		Cytosolic 6-phosphogluconate dehydrogenase	23.6/7	1.21 ± 0.04		−1.39 ± 0.003
226530488		Glucose-6-phosphate 1-epimerase-like	11.9/3	1.28 ± 0.05		/
194688844		Sucrose synthase2	29.5/11	1.61 ± 0.15		/
293333951		Isocitrate dehydrogenase [NAD] Regulatory subunit 1	20.9/4	/		−1.41 ± 0.14
226504732		Sorbitol dehydrogenase homolog1	56.2/27	2.08 ± 0.10		1.39 ± 0.05
226499336		Succinate dehydrogenase flavoprotein subunit	12.9/2	1.45 ± 0.14		/
291047790		Succinate semialdehyde dehydrogenase	23.1/8	1.41 ± 0.12		/
257726331		Cytokinin-*O*-glucosyltransferase 2	7.54/2	1.40 ± 0.04		/
Transcription factor						
226496988		Fibrillarin-2	20.5/2	1.51 ± 0.13		−1.29 ± 0.15
308081068		AP2-EREBP-type transcription factor	12.9/2	1.33 ± 0.06		/
Amino acid and lipid metabolism						
227478191		Argininosuccinate synthase	12.3/3	1.78 ± 0.23		1.27 ± 0.08
293332891		Isovaleryl-CoA dehydrogenase	13.0/2	1.77 ± 0.14		/
195605626		Oleosin 16 kDa	41.0/14	1.32 ± 0.07		−1.30 ± 0.003
Secondary metabolism						
226500722		Anthocyanidin 5,3- *O*-glucosyltransferase	5.86/2	1.23 ± 0.02		−1.33 ± 0.11
293335591		Dihydrolipoyl dehydrogenase	15.0/6	1.32 ± 0.11		/
52699545		Isopentenyl-diphosphate delta isomerase 2	8.72/2	1.47 ± 0.15		/
596080		Thiamine biosynthetic enzyme	16.4/4	1.39 ± 0.12		1.57 ± 0.23
Membrane and transport						
75278333		Aquaporin TIP1-1	6.4/3	1.34 ± 0.09		/
75308033		Aquaporin TIP3-2	7.86/2	1.41 ± 0.05		1.84 ± 0.05
293332063		Cytochrome b5	11.9/2	−1.41 ± 0.11		/
Others and unknown						
226495887		Adaptin ear-binding coat-associated protein 1	12.8/1	1.25 ± 0.04		−1.33 ± 0.17
219363567		Hypothetical protein LOC100217226	9.47/1	−1.71 ± 0.10		−1.35 ± 0.06
219363597		Hypothetical protein LOC100216972	12.0/1	1.32 ± 0.13		−1.77 ± 0.17
226528357		Hypothetical protein LOC100278634	34.8/3	1.59 ± 0.18		/

^a^ GI number in the National Center for Biotechnology Information (NCBI) GenBank database; ^b^ The identified proteins were named according to the annotations in the NCBI database. According to the comments in NCBI, many of these proteins were annotated based on homology evidence (*Arabidopsis*, rice, *etc*.). A protein with identify or significant homology to a known protein was annotated as the same name. A protein without any functional annotations was annotated as a “hypothetical” protein; ^c^ Sequence coverage (%) is calculated by dividing the number of amino acids in the peptide fragments observed by the protein amino acid length; ^d^ Peptide fragments refer to the number of matched peptide fragments generated by trypsin digestion; ^e^ The fold change is expressed as the ration of intensities of up-regulated (positive value) or down-regulated (negative value) proteins between drought stress treatments and control (well water condition). The fold change SD is presented as the mean standard deviation (SD). “/” is used to define no difference or no significant change between drought treatment and the well-watered control.

Based on hierarchical clustering of expressed trends in relative abundance, the differentially expressed proteins were mainly classified into four groups (Figure S1). Increases in abundance may represent specific sensitivity or adaptation of these maize lines to drought stress, whereas a decreases in abundance may reflect cellular damage caused by exposure to drought stress conditions [14]. Our observations documented the variation in abundance of these differentially expressed proteins in response to drought stress and imply that the plants detected the extent of drought-induced stress and alleviated it by modulating the expression of stress-responsive proteins.

### 2.2. Functional Annotation and Categories of Differential Expressed Proteins

To further identify and annotate these proteins, we searched the NCBInr protein database for homologous sequences using BLASTp program [19]. The protein sequences were >40% identical with those of their homologs, suggesting that the proteins might have similar functions. Among the 78 proteins (Table 1), 75 were found in the Pfam and Inter-Pro databases as proteins with putative or known functions, whereas the three remaining proteins (LOC100217226, LOC100216972, LOC100278634) were annotated as unknown or hypothetical proteins.

**Figure 1:**
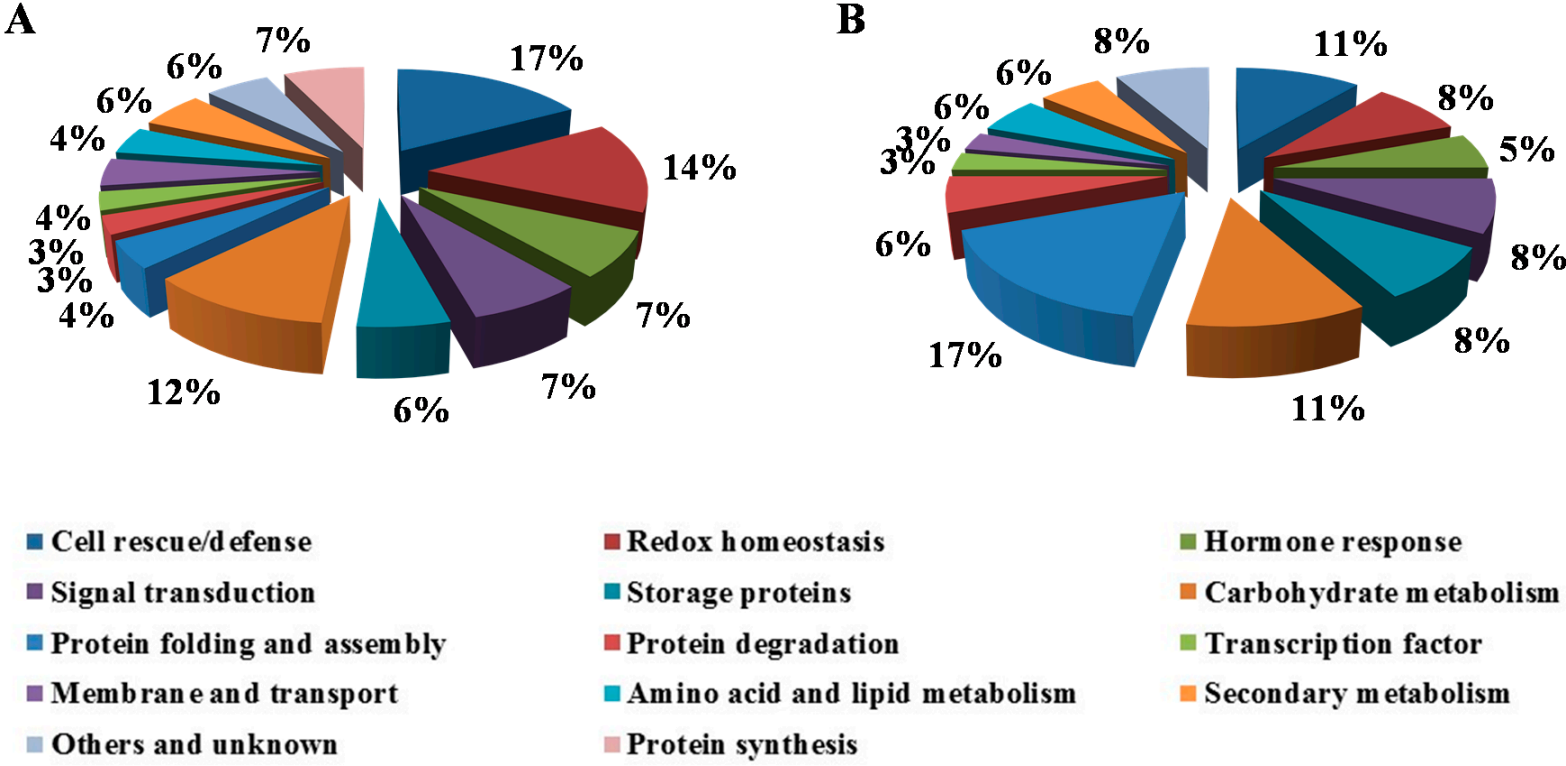
Functional classification of the identified proteins. The pie chart shows the distribution of drought-responsive proteins into their functional classes in percentage. (**A**) Drought-responsive proteins in B73; and (**B**) Drought-responsive proteins in Lo964.

In order to understand the roles of proteins associated with drought-induced changes in the kernels, the differentially expressed proteins were sorted into different categories based on their functions using the Pfam and InterPro databases (Figure 1). In terms of quantitative changes in response to drought stress, proteins involved in cell rescue/defense, redox homeostasis, signal transduction and carbohydrate metabolism account for the majority of differentially expressed proteins in both B73 and Lo964. In B73, the most-represented groups of proteins were comprised of cell rescue/defense (17%), redox homeostasis (14%) and carbohydrate metabolism (12%), followed by protein synthesis (7%), hormone response (7%) and signal transduction (7%). However, the larger groups in the Lo964 were protein folding and assembly (17%), carbohydrate metabolism (11%) and cell rescue/defense (11%), followed by redox homeostasis (8%), signal transduction (8%) and storage proteins (8%). This finding implies that proteins involved in cell rescue/defense and redox homeostasis play important roles in B73 responses to drought, but protein folding and assembly-related proteins may play major roles in Lo964 responses under the same drought conditions.

Further analysis of the abundance changes in each group revealed that these drought stress-related proteins in the main categories were overrepresented, suggesting that these processes were of functional importance for drought stress resistance and adaptation. Similar results were observed by Benešová *et al.* [14], who suggested that drought-induced changes in maize seedling proteome patterns were related to genotype-specific differences, many of which involved proteins within the functional categories mentioned above. In many cases, stresses or injuries can induce plants to modulate protection responses at the biochemical and molecular level. Similarly, drought stress can result in modification of the protein components through changes of gene expression or altered protein synthesis, stability, structural modification or degradation. Such changes can be accompanied by re-homeostasis of various metabolic processes between drought-induced disorder/damage and adjustment, adaptation and maintenance of plants themselves [1,20,21]. This regulatory scenario showed up-regulation of protein biosynthesis and degradation, and following up-regulation of proteins involved in redox homeostasis and cell rescue/defense in B73, but a down-regulated trend of proteins involved in protein folding, assembly and degradation in Lo964.

### 2.3. Overlapping and Specific Proteomic Response to Drought between B73 and Lo964 Lines

**Figure 2:**
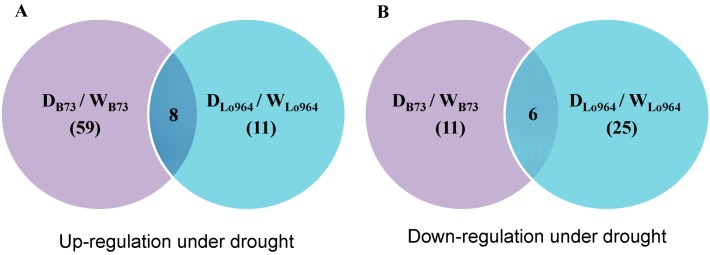
Venn diagram representing the overlap of the identified proteins in abundance with or without drought stress treatments in drought-tolerant maize line Lo964 and drought-sensitive maize line B73. (**A**) Up-regulation of proteins under drought stress; and (**B**) Down-regulation of proteins under drought stress.

Following field drought treatments, 70 proteins were differentially expressed in B73; of these, 59 proteins were up-regulated and 11 were down-regulated (Figure 2). In Lo964, 36 proteins were differentially expressed; of these, 11 proteins were up-regulated and 25 were down-regulated (Figure 2). In addition, 14 proteins showed similar responsive patterns (eight up-regulated and six down-regulated) in both lines under drought stress. These common up- or down-regulated proteins were mainly distributed in the functional categories of redox homeostasis, cell rescue/defense, hormone response, signal transduction, carbohydrate metabolism and protein degradation. Thirteen differentially expressed proteins were up-regulated in B73 and down-regulated in Lo964; however, only histidine kinase was down-regulated in B73 and up-regulated in Lo964 (Table 1).

The drought-induced proteome patterns showed that field drought stress induced up-regulation of more proteins in B73 than in Lo964, whereas the reverse was true for down-regulated proteins. This difference was particularly obvious in the case of proteins involved in the regulation of cell rescue/defense, redox homeostasis and protein metabolism, indicating that the differential drought sensitivity of the examined lines is associated with changes in the expression of proteins with similar functions. These differences in expression were similarly reflected in data on stress-related and pathogenesis-related gene expression under drought stress in the two examined lines based on our previous studies [16,22,23,24,25]. These results imply that the two examined maize lines used different strategies to cope with drought stress during kernel development. In addition, *A. flavus* infection of maize kernel also induced similar changes in these functional protein groups [26,27,28]. It is proposed, therefore, that drought stress treatment of drought tolerant maize lines may improve resistance to *A. flavus* through enhancing of defense/stress-related protein expression [27].

### 2.4. Gene Ontology Analysis of Drought-Responsive Proteins

To gain further knowledge of biological functionality of the drought-responsive, differentially expressed proteins in Lo964 and B73 kernels, the proteins from each line were analyzed separately by Gene Ontology in the PIR (Protein Information Resource) database with three sets of ontologies: biological process (GO-BP), molecular function (GO-MF) and cellular component (GO-CC). As shown from the results of GO-BP analysis, drought-responsive proteins were involved in diverse biological processes in both Lo964 and B73.

For drought-responsive proteins in Lo964, the most highly-enriched GO-BP categories were cell rescue/defense and protein biosynthetic processes (35.4%), demonstrating that these processes were of functional importance for drought responses in Lo964. The second highly-enriched GO-BP categories were metabolic processes and storage proteins (17.6%) (Figure S2A). As for the GO-MF categories, binding activity (including protein binding), ATP binding, RNA binding and GTP binding contributed the biggest portion of the proteins (38.7%), with dehydrogenase activity second (13%) (Figure S2C). For the GO-CC ontology, the top two categories were cytoplasm (38.1%) and nucleus (26.2%) (Figure S2E).

For drought responsive proteins in B73, the top two GO-BPs were oxidation-reduction processes and stress responses (27.7%); and the second was defense response, hormone-activated signaling and translation (24.6%) (Figure S2B); whereas, compared with Lo964, the composition of GO categories in B73 also changed significantly. The top three GO-MFs were binding activity, including ATP binding, GTP binding and protein binding (31.4%), transferase activity (10.0%) and ribosomal structural constituents (8.6%) (Figure S2D). Finally, the top two GO-CCs were cytoplasm (27.1%) and nucleus (15.7%) (Figure S2F). The results of the GO analyses indicate that the drought responsive proteins in Lo964 and B73 had similar distributions in GO-BP, GO-MF and GO-CC, yet some important differences still exist, such as a higher proportion of biosynthetic processes in Lo964 and a higher proportion of oxidation-reduction process responses in B73. Therefore, although drought stress greatly affected stress defense and metabolism-related pathways, their subordinate processes in Lo964 and B73 could be different and involve various sets of responsive proteins with different biological and molecular functions, as well as different sub-cellular localizations.

### 2.5. Sensitivity to Drought Stress Is Characterized by the Up-Regulation of Antioxidative Proteins

**Figure 3:**
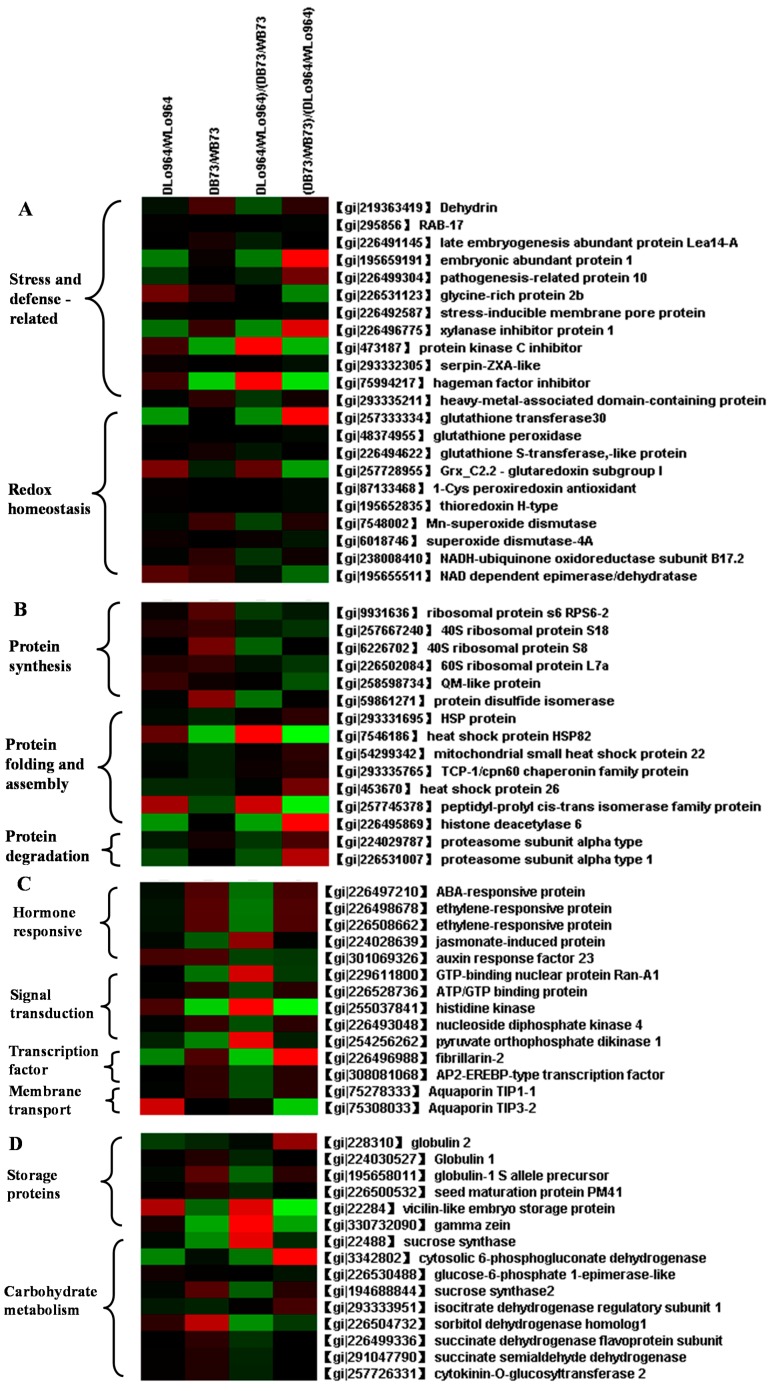
Hierarchical clustering of drought stress responsive proteins involved in cell rescue/defense and redox homeostasis (**A**), protein metabolism (**B**), hormone responsive, signal transduction, transcription factor and membrane transport (**C**) and carbohydrate metabolism and storage proteins (**D**). Hierarchical cluster analysis was conducted using the Cluster 3.0 (University of Tokyo, Tokyo, Japan) and Treeview software (University of Glasgow, Scotland, UK).

The most represented functional category of proteins responding to drought was antioxidative proteins. A total of 22 identified proteins are reported to function in antioxidative reactions and were classified into two functional categories: cell rescue/defense and redox homeostasis (Figure 3A). Of the 12 proteins classified as cell rescue/defense-related proteins, 10 (dehydrin, RAB-17, late embryogenesis abundant protein (LEA14-A), embryonic abundant protein 1, PR-10, glycine-rich protein 2b, stress-inducible membrane pore protein, xylanase inhibitor protein 1, serpin-ZXA-like, heavy-metal-associated domain-containing protein) were up-regulated and two (protein kinase C inhibitor, and Hageman factor inhibitor) were down-regulated in B73 under drought stress, whereas Lo964 was characterized only by up-regulation of the glycine-rich protein 2b and down-regulation of the embryonic abundant protein 1, PR-10 and xylanase inhibitor protein 1 (Table 1; Figure 3A).

Dehydrins (DHNs) and the members of Group II late embryogenesis abundant proteins (LEAs), along with LEA14-A and glycine-rich protein 2b, play a fundamental role in plant response and adaptation to drought stress [29]. The expression of these hydrophilic, glycine-rich proteins is also known to be induced under dehydration in sensitive genotypes of other plant species, such as Bermuda grass and rice [29,30]. Their expression also contributes to increased tolerance to ABA accumulation and drought stress in maize [31,32]. In particular, DHNs are important for preserving the stability of membrane-associated proteins and macromolecules, the adjustment of cytoplasmic osmotic pressure and the prevention of cell protein denaturation by the binding of water molecules to their surfaces [33]. These proteins showed increasing patterns of expression in B73 in response to drought conditions. Moreover, the genes encoding these proteins displayed corresponding increasing trends in response to drought stress in B73 and Lo964 kernels at the transcriptional level [23]. PR-10 is a member of the PR protein family that potentially functions in plant defense to abiotic or biotic stress [34,35], and was found to be up-regulated in B73 and down-regulated in Lo964 under drought stress in this study. Xylanase inhibitor protein 1, protein kinase C inhibitor and the Hageman factor inhibitor are natural defense-related proteins present in seeds and are induced in certain plant tissues by pathogen infection, herbivory or wounding [34,36]. It was unexpected that these three proteins would be down-regulated in response to drought stress, but a possible explanation may be that the scope of their functionalities under drought stress has yet to be described in the literature. The question of how these cell rescue/defense-related proteins are implicated in maize kernel antioxidative reactions still remains to be addressed.

Plants characteristically respond to drought stress by the rapid production of reactive oxygen species (ROS), including superoxide radicals (O_2_^–^), hydrogen peroxide (H_2_O_2_) and hydroxyl radicals (OH), which can perturb cellular redox homeostasis and result in oxidative damage to many cellular components and structures along with over-reduction of electron transport chain components in the mitochondria, plastids and various detoxification reactions [37]. This also results in an imbalance between ROS and the antioxidative defense system (AOS). Therefore, a fine regulation of the steady state and responsive signaling levels of ROS in plants is necessary to avoid injury and to maintain an appropriate level by which different developmental and environmental signals can be perceived and transmitted [38]. In this study, ten of the identified proteins are implicated in redox homeostasis-related functions, including: glutathione transferase 30 (GT), glutathione *S*-transferase (GST), glutaredoxin (Grx), thioredoxin H-type (Trx), peroxiredoxin (Prx), two isoforms of superoxide dismutase (SOD), NADH-ubiquinone oxidoreductase subunit B17.2 and a NAD-dependent epimerase/dehydratase family protein (Table 1 and Figure 3A). These proteins are involved in the glutathione-ascorbate cycle (including GPX and Grx), the peroxiredoxin/thioredoxin (PrxR/Trx) pathway (including Trx, Prx and UOR), the glutathione peroxidase (GPX) pathway (including GPX, Grx, GST and GT) and superoxide dismutation (including SOD), respectively (Figure S3). Therefore, it is clear that that the ROS scavenging system may be activated in maize kernels to alleviate such oxidative damage and to enhance drought tolerance. Two enzymes, glutathione peroxidase (GPX) and Grx_C2.2-Glutaredoxin subgroup I (Grx), which are involved in the glutathione-ascorbate cycle for removing H_2_O_2_, showed the same expression patterns (Figure 3A and Figure S3). GST and GT can reduce H_2_O_2_ to its corresponding hydroxyl compounds to remediate oxidative membrane damage, and their expression is strongly enhanced by biotic and abiotic stresses [39]. NADH-ubiquinone oxidoreductase (UOR), 1-Cys peroxiredoxin and thioredoxin (H-type) also play an important role in the peroxiredoxin/thioredoxin-based redox pathway as components of the antioxidative defense system [40]. These proteins were up-regulated in response to drought stress in B73. Compared with B73, the status of redox homeostasis in Lo964 was subtly influenced by drought treatment, in which Grx and UOR showed increases in abundance to a different degree and GT was down-regulated in response to drought (Figure 3A and Figure S3). Some of these proteins were consistent with those observed in the maize line, Tex6, in response to drought in developing kernel tissues [15]. Thus, our results, in conjunction with those previously reported [14,15,23,41], show that the up-regulation of these proteins implies that the antioxidative defense system is induced, and the ROS scavenging system was activated by the severe drought stress response in B73 in order to alleviate oxidative stress. In contrast, the stress response was mild in Lo964, resulting in higher ROS scavenging system expression in B73 than in Lo964 (Figure S3).

### 2.6. The Response to Drought Stress May Be Connected to the Differential Regulation of Protein Metabolism

The differential responses of the two examined lines to drought stress were also observed to be involved in protein metabolism, which plays a pivotal role in drought response [42] (Table 1). Seventeen observed, differentially expressed proteins are involved in protein metabolism and were divided into three functional groups (Figure 3B). The first group consists of five identities functioning in protein biosynthesis with up-regulated trends only in B73, including RPS6-2, 40S ribosomal proteins S18 and S8, 60S ribosomal protein L7a and a QM-like protein, all of which are directly involved in the initiation of newly synthesized peptide chains. The second group involves eight identities related to protein folding and assembly, including four HSPs: TCP-1, a peptidyl-prolyl *cis*-*trans* isomerase family protein, histone deacetylase 6 and protein disulfide isomerase. These HSPs and chaperonins have been well studied and are known to be responsible for protein refolding and assembly in the abiotic stress response [42]. As illustrated in Figure 3B, there is an interesting trend in the expression of these proteins that are responsible for protein folding and assembly, respectively. Drought stress remarkably decreased the expression levels of the highly conserved, stress-related HSPs and chaperonins in B73, but caused a varying expressed patterns in Lo964, suggesting that protein refolding and assembly may be inhibited in this sensitive line under drought stress. The third group in this category has two members of α type proteasome subunits associated with protein degradation (Figure 3B). Interestingly, the opposing expression patterns of the two proteasome subunit isoforms observed in Lo964 and B73 may imply that they may possess different roles in responding to drought stress and can regulate both signal transduction and transcription in response to environmental stresses [43].

Taken together, the expression patterns of proteins in this functional category imply that enhancement of protein biosynthesis and protein degradation in B73 and inhibition of protein folding, assembly and degradation in Lo964 are required for responding to drought stress conditions, and a self-regulated and adapted system is crucial for genotype-dependent drought stress accommodation (Figure S4).

### 2.7. Differentially Expressed Proteins Implicated in Hormone Response and Signal Transduction Pathways

When the lines were exposed to field drought conditions, ten proteins were differentially expressed that function in phytohormone responses and signal transduction pathways (Table 1). Among those, five proteins (ABA-responsive protein, two isoforms of ethylene-responsive protein, auxin response factor 23 and a jasmonate-induced protein) were related to the hormone response, inferring that drought stress initiated hormone signaling events. ABA- and ethylene (ET)-responsive proteins were up-regulated in B73 under drought conditions, but not in Lo964. Auxin response factor 23 showed up-regulations, and jasmonate-induced protein showed down-regulation in both B73 and Lo964 under drought stress (Table 1; Figure 3C). These results imply that ABA, ET, auxin and JA regulate protective responses in maize against drought stress through synergistic and antagonistic actions, which are collectively referred to as “signaling crosstalk” [44,45,46].

It has been reported that severe drought stress can increase the production of ABA and ET and subsequently up-regulate the expression of ABA or ET-responsive proteins [47]. ABA, ET and interactions between these two stress-induced hormones control many of the responses of plant to drought stress [48], and it has also been proposed that both ABA and JA participate in responses to moderate drought stress in *Arabidopsis thaliana* [44]. Our previous studies also showed increases in ABA and IAA content in maize kernels (lines Lo964 and Lo1016) in response to drought stress [23]. Aimar *et al .* [45] showed that JA levels were not significantly affected in *Panicum virgatum* (switchgrass) during drought treatments. However, after water was restored, JA content consistently increased to levels greater than that of the well watered control. Subsequently, plants naturally produced stress hormones that signal adverse conditions and helped them adapt to the drought environment [45].

Of five signal transduction-related proteins, two identities (GTP-binding nuclear protein Ran-A1 and pyruvate orthophosphate dikinase 1) showed downregulated expression patterns in two both lines, whereas ATP/GTP binding protein and nucleoside diphosphate kinase 4 were increased in abundance in B73, but not in Lo964 under drought stress (Table 1 and Figure 3C). It is known that GTP-binding nuclear protein Ran-A1 and ATP/GTP binding protein, members of the GTP-binding family of proteins, participate in small GTPase-mediated signal transduction pathways that are activated by external signals [49]. The different expression patterns of the GTP binding proteins suggest that each member might participate in a different signaling pathway. In addition, pyruvate orthophosphate dikinase (PPDK) is a key enzyme in gluconeogenesis and photosynthesis that is responsible for reversing the reaction performed by pyruvate kinase in Embden–Meyerhof–Parnas glycolysis, and the expression of maize-specific PPDK in rice has been shown to enhance drought tolerance [50]. Therefore, the up-regulation of ATP/GTP binding protein and nucleoside diphosphate kinase 4, together with the down-regulation of Ran-A1 and pyruvate orthophosphate dikinase 1, suggests that different signaling systems are initiated in drought-treated kernels of Lo964 and B73.

### 2.8. Differentially Expressed Proteins Are Involved in Carbohydrate Metabolism and Storage

Proteins associated with carbohydrate metabolism also showed altered expression patterns induced by drought stress. Nine of the identified proteins are involved in carbohydrate metabolism, including two isoforms of sucrose synthases, cytosolic 6-phosphogluconate dehydrogenase, glucose-6-phosphate 1-epimerase-like protein, isocitrate dehydrogenase [NAD] regulatory subunit 1, mitochondrial-like sorbitol dehydrogenase homolog 1, succinate dehydrogenase flavoprotein subunit, succinate semialdehyde dehydrogenase and cytokinin-*O*-glucosyltransferase 2 (Table 1). Among them, one membrane-bound sucrose synthase isoform showed a decreased abundance under drought stress in B73 and Lo964, but the other isoform, sucrose synthase 2, showed an increased abundance in B73 (Figure 3D). It was reported that sucrose synthase 2 was found to be involved in wheat grain responses to drought stress [51]. The succinate dehydrogenase flavoprotein subunit and succinate semialdehyde dehydrogenase are two components that participate in both the citric acid cycle and the electron transport chain. Both showed up-regulated trends in B73 under drought stress, but no changes in Lo964. The transcription of succinate dehydrogenase flavoprotein subunit has been found to be up-regulated in *Ilex paraguariensis* leaves in response to water deficit and ABA application [52]. In addition, drought also induces changes in the carbohydrate content of several plant species, which may result in altered expression of proteins involved in carbohydrate metabolism [53,54]. The changes of protein expression patterns related to carbohydrate metabolism were essential for the adjustment and feedback that plants make under drought stress, because carbohydrate metabolism is the hub of the main bio-molecular metabolism, and the substrates involved in carbohydrate metabolism provide the essential energy that the plant needs.

As expected, the expression of proteins involved in the category of storage proteins was influenced by drought stress in developing maize kernels. Six proteins were found to be altered in abundance, including globulin 1, globulin-1 *S* allele precursor, globulin 2, seed maturation protein PM41, vicilin-like embryo storage protein and γ-zein (Table 1). Among them, there are three proteins (globulin 1, globulin-1 *S* allele precursor and seed maturation protein PM41) that were up-regulated in B73 under drought treatment, but only one protein, vicilin-like embryo storage protein, was up-regulated in Lo964 (Figure 3D). In addition, γ-zein displayed down-regulated expression in both B73 and Lo964. These results further demonstrated that the biochemical balance involved in protein synthesis, folding, assembly and degradation were disordered under drought conditions in the developing kernels of the two lines in this study.

### 2.9. Drought Response on Other Functional Groups

Other proteins identified in the present study are membrane-associated and transport proteins, transcription factors and others that are involved in amino acid and lipid metabolism, secondary metabolism and other unknown functions (Table 1). It is suggested that drought stress influenced these physiological and biochemical processes in both Lo964 and B73. Among these identified proteins, aquaporins (TIP1-1 and TIP3-2), which are integral membrane pore proteins that serve to facilitate the transcellular symplastic pathway for water transport [55], accumulated at a higher level in Lo964 under drought stress, but not in B73. This may imply that Lo964, which was characterized by a larger root system, is likely to efficiently adjust the transport of water, allowing it to be more tolerant to drought stress conditions.

### 2.10. Protein–Protein Interaction Analysis

**Figure 4:**
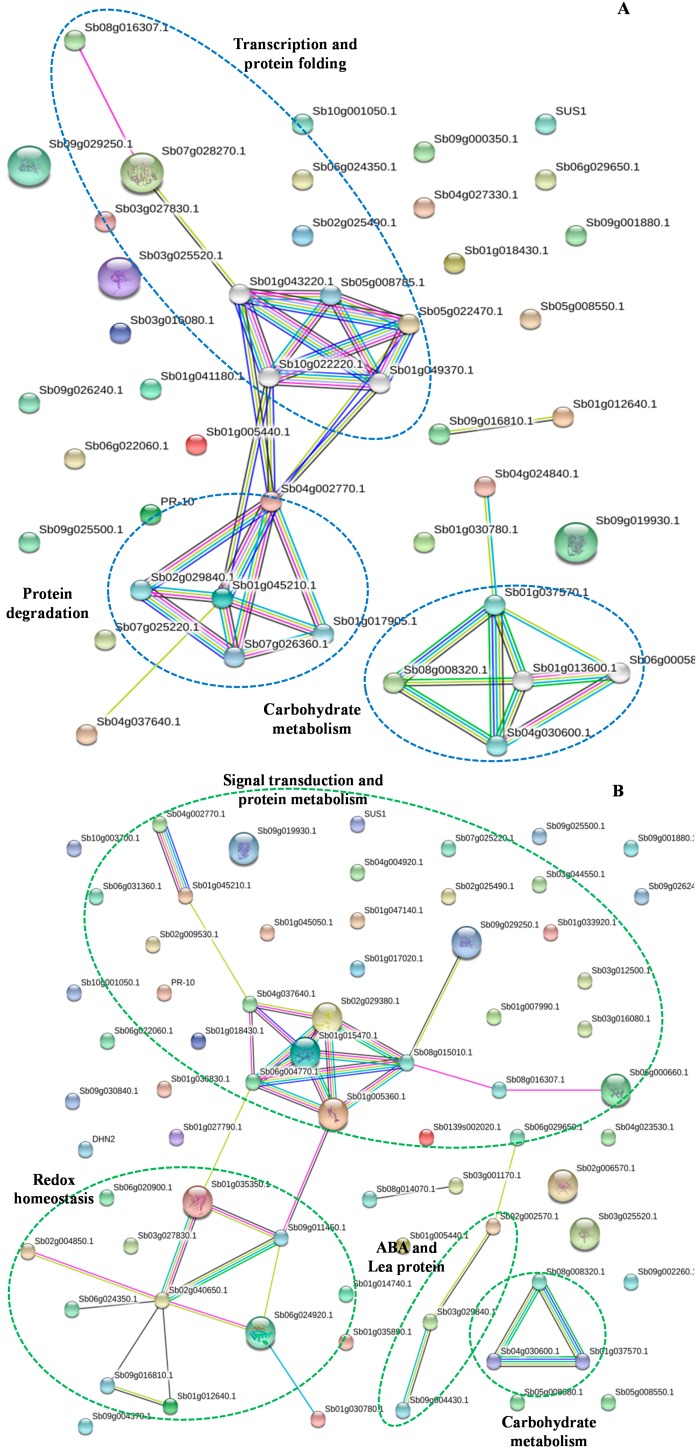
Protein-protein interaction networks analyzed by String software. (**A**) Network analyzed from drought responsive proteins in Lo964 under drought stress condition; (**B**) Network analyzed from drought responsive proteins in B73 under drought stress condition. Different line colors represent types of evidence for association: Green line, neighborhood evidence; pink line, fusion evidence; purple line, experimental evidence; light blue line, database evidence; black line, coexpression evidence; blue line, co-occurrence evidence; and yellow line, text-mining evidence. The networks indicated by broken oval shapes represent the functional modules.

Proteins in plant cells and tissues do not act as single molecules, but rather play interrelated roles together in the context of networks. To determine how the maize plants transmit drought stress signaling through protein–protein interactions to affect cell functions in Lo964 and B73, the proteins identified as being significantly regulated were analyzed using the String database. Some of the drought responsive proteins were predicted to interact with each other, and networks of interaction were constructed *in silico* (Figure 4). The abbreviations of the protein names in the networks are shown in Table S1. Two groups of protein interactions were detected in Lo964, including FIB2-HDAC6-HSP-TCP-TCP-1-PSMA1-PSMA and NAD-IDH-SuS-SuS2-AS (Figure 4A). However, three groups of protein-protein interactions were detected in B73, including GBP-FIB2-RPS6-2-RP-L7a-QM-RP-S18-RP-S8-HSP82-HDAC6-SMP-PM41-Serpin-ZXA-PSMA1-PSMA-MnSOD-SOD-4A-GPX-1-cysPrx-GT30-Trx H-v-ESP-EAP1-Oleosin 16, ABR-Lea14-A and AS-SuS2-G6P1E-IVD-DLD-SMP-PM41-SDH-GRP2b (Figure 4B). These results show two partly overlapping, but distinct networks in Lo964 and B73. The proteins comprising the interaction networks have important functions in defense responses, redox homeostasis, signal transduction and protein and carbohydrate metabolism. Two groups of protein networks involved in GBP-FIB2-RPs-HSP-PSMA and NAD-IDH1-SUS-AS were commonly regulated in B73 and Lo964 in response to drought. However, the interactions made up of antioxidative enzymes coupled with signal transduction and protein metabolism occupied a central position and may play an important role in responses to drought stress in B73.

The connectivity of proteins in these interaction networks can also provide insight into their relative importance in biological processes. Protein “hubs” (connected to many other proteins) and “bottlenecks” (key connectors of sub-networks), such as FIB2 and SOD in B73 and TCP-1 in Lo964, represent central points for controlling communication within the network and tend to play essential roles in responses to drought stress. In addition, the nodes that are not connected with other proteins within interaction networks indicate that those proteins did not interact with other proteins based on the STRING database analysis and may play indirect roles in maize responses to drought stress.

### 2.11. Models for Maize Kernels Tolerance and Sensitivity to Drought Stress

**Figure 5:**
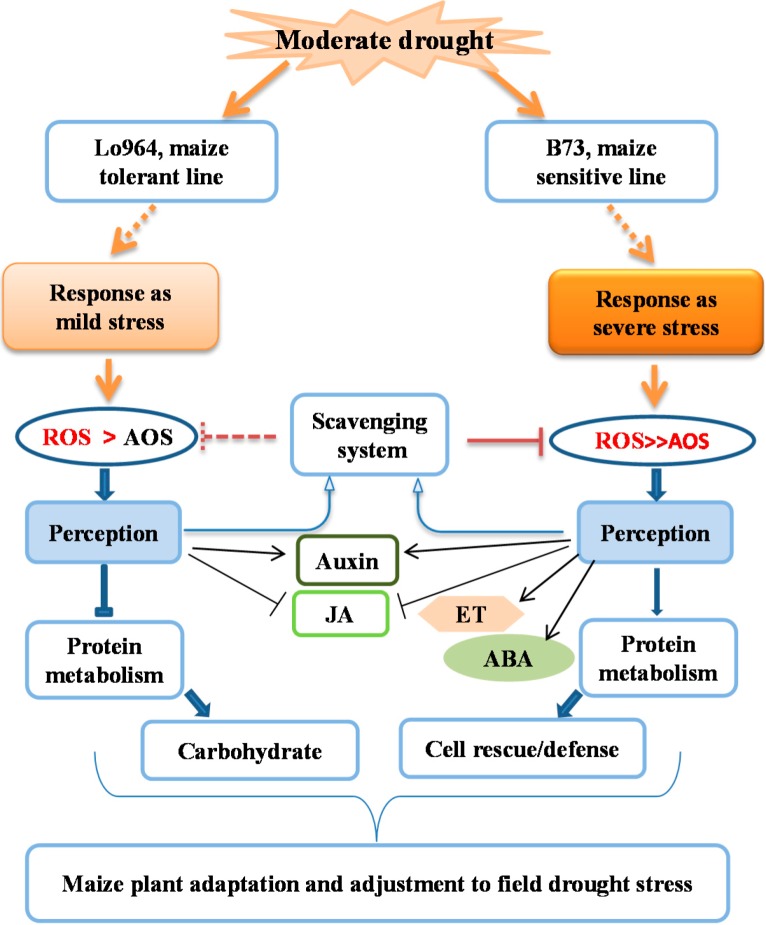
A schematic of drought stress responses in kernels of the two examined maize lines, Lo964 with an extensive, deep and strong lateral root system [16,56] and B73 with a superficial, fewer lateral root system [18,59].

Plant resistance to drought relies on diverse adaptive strategies, including the regulation of morphology, physiology, biochemistry and molecular genetics. Here, we have demonstrated that resistance and adaptation to drought stress is associated with coordinated and ordered expression of proteins involved in diverse defense signaling pathways and altered substrate metabolism. Based on the described comparative analyses of drought responsive proteins, as well as the sequential events in B73 and Lo964 under drought stress, we propose a comprehensive model to illustrate the cellular events leading to drought resistance or adaptation (Figure 5). In B73, changes in or activation of ABA, ET, auxin and JA signaling, ROS scavenging, protein biosynthesis and protein degradation were the major cellular activities observed. In particular, up-regulated expression of anti-oxidative enzymes in the ROS scavenging system enhanced the defense response of B73 to drought stress. Diverse hormone signaling and accelerated biosynthesis and degradation of proteins also showed more intensive reactions that were initiated by severe drought stress in B73.

In contrast to B73, a more moderate response processes occurred in Lo964, which displayed increased activities of aquaporins, inhibition of protein folding, assembly and degradation and increased carbohydrate metabolism. There may be a possibility that since Lo964 is characterized by an extensive and deep root system, it can readily alleviate the water deficit and drought damage [56]. Differences in root architecture can affect water uptake and flow according to changing water availability, and roots can be considered as sensors that detect changes of water availability in the soil and influence resistance to drought at the whole plant level [47,57]. Moreover, drought tolerance traits have a dual effect, positive in very severe scenarios and negative in milder scenarios, or *vice versa* [58]. It is better understood that field drought conditions resulted in a severe drought stress in B73, which possesses a superficial yet extensive root system [59], and the same field drought treatments caused increased expression of proteins involved in ROS scavenging in B73 compared to Lo964. The excessive ROS caused an imbalance of the original redox homeostasis, which would lead to more changes in signaling pathways and molecular metabolism. In addition, the proteins of auxin signaling activation and JA signaling were down-regulated between the two examined genotypes, presumably facilitating the homeostasis of diverse hormone signaling pathways. The activating order of these signaling events and cross-talk between them could be critical in forming resistance or adaptation to different drought stress scenarios [58]. Generally, these changes of metabolic processes and status eventually lead the plants to a new homeostasis that can adapt to and/or resist external adverse stresses. Such a protein network can help us understand the possible regulatory strategy occurring in drought-treated maize kernels.

The field drought treatments resulted in higher numbers and levels of differentially expressed proteins in B73 compared to Lo964, which contrasts with previously observed scenarios in which more defense and stress response proteins accumulated in maize seedling leaves of drought tolerant lines compared to susceptible ones [14]. However, additional studies examining gene expression in seedling leaf tissues of maize and cereal crops have indicated greater levels of expression in response to drought in susceptible lines than in resistant lines. For example, Zheng *et al.* [60] showed that a relatively smaller number of genes were induced in transcriptome analysis of maize seedling leaf tissues of the tolerant maize line, Han21, than in the sensitive Ye478 line under drought stress. Furthermore, Kausar *et al.* [61] detected a higher number of differentially expressed proteins in barley seedling leaves of sensitive lines than in the tolerant ones under drought treatments. Therefore, it is possible that field-based drought treatments, different genetic backgrounds or tissue-specific responses can affect the results of expression and proteomic studies in cereal drought responses.

A similar phenomenon to that observed in the present study was observed in response under *A. flavus* infection in kernel and rachis tissues [27,62]. Chen *et al.* [27] detected that more resistance-related proteins were up-regulated in maize kernel endosperm in resistant lines compared with susceptible ones, while Pechanova *et al.* [62] found that maize-resistant rachis relies on constitutive defenses, while susceptible rachis is more dependent on inducible defenses under infection of the maize with *A. flavus*. The accumulation of such stress-related proteins may afford not only resistance to *A. flavus* colonization and aflatoxin contamination, but may also provide drought stress tolerance in maize tissues [7]. Of course, all of these results and conclusions will need to be further confirmed and elaborated in future research.

## 3. Materials and Methods

### 3.1. Plant Materials and Growth Conditions

Two maize (*Zea mays* L.) inbred lines, the drought-tolerant line, Lo964, and drought-sensitive line B73, were selected for use in this study. The two inbred lines were grown in field rain-out shelters in the normal maize growing season from April to August at Bellflower Farm, USDA-ARS (Agricultural Research Service, Corvallis, OR, USA), CPMRU and Tifton Campus of the University of Georgia, which were divided into two regions for well-watered control and drought stress treatments. At 14 DAP, the plants in control shelters were watered normally, whereas plants in the drought treatment were subjected to drought simulation by withholding water to 50% of applied irrigation in the well-watered control. The environmental conditions were recorded using a Watchdog weather station (Spectrum Technologies, Aurora, IL, USA). During the drought treatment period, the temperature ranged from 28 to 38 °C during the day and from 19 to 26 °C at night; and the relative humidity ranged from 81% to 97% during the day and from 45% to 79% at night inside the rain-out shelters. Maize kernels were collected from both treatments at 35 DAP, immediately frozen in liquid nitrogen and subsequently stored at −80 °C prior to proteome analysis. The experiment was repeated twice in 2009 and 2010 with two replicates for each line in each year.

### 3.2. Protein Extraction, Digestion, iTRAQ (Isobaric Tags for Relative and Absolute Quantitation) Labeling

Total proteins were extracted from kernels of control and drought-stressed plants according to previously described procedures [63,64]. The concentration of each extract was determined using a Bradford protein assay kit (Bio-Rad, Hercules, CA, USA) with bovine serum albumin (BSA) as a standard. Furthermore, tricine-sodium dodecyl sulfate polyacrylamide gel electrophoresis (Tricine-SDS-PAGE) was used to verify the quantitative results from the Bradford assay and determine the quality of the extract [65].

Protein extracts (100 µg) were thawed and precipitated with acetone overnight. After precipitation, the pellets were dissolved in 1% SDS containing 100 mM triethylammonium bicarbonate (pH 8.5), then reduced using 50 mM dithiothreitol, then alkylated using 250 mM iodoacetamide and finally digested using trypsin (Promega, Madison, WI, USA). The proteins were then labeled using an iTRAQ Reagents 8-plex kit according to the manufacturer’s instructions (Applied Biosystems, Warrington, UK). The control replicates of Lo964 were labeled with iTRAQ tags 113 and 117. The drought-stressed replicates were labeled with tags 114 and 118. The control replicates of B73 were labeled with iTRAQ tags 115 and 119, and the drought-stressed replicates were labeled with tags 116 and 121. No significant differences in labeling efficiency among these isotope tags were observed in this study. After labeling, the samples were mixed and aliquoted into four technical repeats.

### 3.3. Analysis of Strong Cation Exchange Fractionation, Reverse Phase Nanoflow HPLC and Tandem Mass Spectrometry

The peptide fractionation and analysis of tandem mass spectrometry were conducted as previously described [66,67]. The combined peptide mixtures were dried and dissolved in Solvent A (25% (*v*/*v*) acetonitrile, 10 mM ammonium formate, pH 2.8). The peptides were fractionated on an Agilent HPLC system 1100 (Agilent Technologies, Waldbronn, Germany) using a polysulfoethyl A column (2.1 × 100 mm^2^, 5 µm, 300 Å; PolyLC, Columbia, MD, USA) according to the User’s Guide. Peptides were eluted at a flow rate of 200 µL/min with a linear gradient of 0%–20% Solvent B (25% (*v*/*v*) acetonitrile, 500 mM ammonium formate) over 60 min followed by ramping up to 100% Solvent B over 5 min and holding for 20 min. Absorbance at 214 nm was monitored, and a total of 10 fractions were collected. Each strong cation exchange (SCX) fraction was subjected to reverse phase nanoflow HPLC separation and quadrupole time-of-flight (QSTAR XL) mass spectrometry analysis.

### 3.4. Protein Identification and Relative Quantification

Protein identification was also conducted as previously described [66,67,68]. The MS/MS data were submitted for sequence query using the ProteinPilot (version 2.0.1) software (Applied Biosystems, Foster City, CA, USA) against a local maize protein database downloaded from the National Center for Biotechnology Information (NCBI) (171,626 sequences, downloaded on 31 October 2013). Fixed modification of methyl methane thiosulfate-labeled cysteine, methionine oxidation, fixed iTRAQ modification of free amine in *N* termini and lysine residues and variable iTRAQ modifications of tyrosine were considered. Parameters, such as trypsin digestion, precursor mass accuracy and fragment ion mass accuracy, are built-in default settings of the software. The false discovery rate (FDR) analysis was performed using the Proteomics System Performance Evaluation Pipeline (Applied Biosystems, Foster City, CA, USA) integrated with the ProteinPilot [69,70], which provides an assessment of the quality of the results, and the detected proteins at the local FDR 5.0% level was used to further conduct comparative proteomic analysis (Table S3).

Protein relative quantification by iTRAQ was performed using the default settings of software. After data normalization, the mean, SD and *p*-values to estimate statistical significance of the changes in protein levels were calculated by the ProGroup™ algorithm of ProteinPilot software (Applied Biosystems, Foster City, CA, USA). For the identification of expression differences, each experimental run was initially considered separately. To be identified as being differentially expressed, a protein had to be quantified with a *p*-value <0.05, and a fold change >1.2 or <0.83 in at least one experimental replicate and one maize line [38,71,72,73].

### 3.5. Bioinformatics Analysis

The functions of the identified proteins were assigned using the protein-function database Pfam [74] and the InterPro database [75,76,77]. Identified proteins were categorized by their functional annotation as described for *Arabidopsis* [78]. Among the identified proteins annotated as either unknown or hypothetical proteins, their sequences were used as queries to search for homologues with BLASTp to annotate these identities [19]. These proteins shared more than 40% positive identity with their corresponding homologues at the amino acid level, suggesting that they might have similar functions with the corresponding homologues with the highest similarity.

The differentially expressed proteins were also used to perform gene ontology (GO) analysis using the PIR database [79] and were assigned to three GO vocabularies: biological processes, molecular function and cellular component. The protein-protein interaction network was analyzed using the STRING program [80] based on genomic context, high-throughput experiments and co-expression experiments in plants.

## 4. Conclusions

The results of this research showed the overlapping and specific proteomic responses to field drought stress in sensitive and tolerant maize lines. Under field drought conditions, proteins involved in redox homeostasis, cell rescue/defense, signal transduction, metabolism and other functional groups showed significant changes in both lines, which suggested that drought influenced these biological processes in both B73 and Lo964. The oxidative burst was more pronounced, and antioxidative systems were further stimulated in the B73 as compared to Lo964. We observed an increase in abundance of proteins involved in protein metabolism in B73, while a decrease in abundance of these proteins was observed in Lo964. In addition, there was also an increase in proteins associated with carbohydrate metabolism, specifically energy reservation and production, which suggests the possible involvement of more energy consumption for self-protection of B73 from drought-related damage. Together, these observations may reveal protein markers or targets that will be useful in future evaluation and assessment of germplasm or in crop protection strategies. It would be interesting to examine how they are coordinated in drought-induced molecular events in future studies.

## Supplementary Materials

Supplementary materials can be found at http://www.mdpi.com/1422-0067/15/10/18892/s1.

## Supplementary Files

Supplementary File 1
