# Supplementary material for: Protein Profiles Reveal Diverse Responsive Signaling Pathways in Kernels of Two Maize Inbred Lines with Contrasting Drought Sensitivity
Source: Int J Mol Sci. 2014 Oct 20;15(10):18892–918. doi: 10.3390/ijms151018892 (PMC4227252; doi:10.3390/ijms151018892)

Supplementary Information

Table S1. The abbreviations of the specific protein names in the protein-protein interaction networks.

Abbreviation	Accession Number in Sorghum Bicolor	Protein Name
1-Cys Prx	Sb02g040650.1	1-Cys peroxiredoxin antioxidant
ABR	Sb08g014070.1	ABA-responsive protein
AS	Sb01g037570.1	Argininosuccinate synthase
DLD	Sb09g004430.1	Dihydrolipoyl dehydrogenase
EAP1	Sb09g016810.1	Embryonic abundant protein 1
FIB2	Sb04g037640.1	Fibrillarin-2
G6P1E	Sb06g031360.1	Glucose-6-phosphate 1-epimerase-like
GBP	Sb09g029250.1	GTP-binding nuclear protein Ran-A1
GPX	Sb06g024920.1	Glutathione peroxidase
GRP 2b	Sb06g029650.1	Glycine-rich protein 2b
GT30	Sb01g030780.1	Glutathione transferase30
HDAC6	Sb08g016307.1	histone deacetylase 6
HSP	Sb07g028270.1	HSP protein
HSP82	Sb06g000660.1	Heat shock protein HSP82
IVD	Sb09g002260.1	Isovaleryl-CoA dehydrogenase
Lea14-A	Sb03g001170.1	Late embryogenesis abundant protein Lea14-A
MnSOD	Sb09g011450.1	Mn-superoxide dismutase
NAD-IDH1	Sb04g024840.1	Isocitrate dehydrogenase [NAD] regulatory subunit
Oleosin 16	Sb06g024350.1	Oleosin 16
PSMA	Sb01g045210.1	Proteasome subunit alpha type
PSMA1	Sb04g002770.1	Proteasome subunit alpha type 1
QM	Sb01g015470.1	QM-like protein
RP- L7a	Sb02g029380.1	60S ribosomal protein L7a
RP-S18	Sb01g005360.1	40S ribosomal protein S18
RPS6-2	Sb08g015010.1	Ribosomal protein s6 RPS6-2
RP-S8	Sb06g004770.1	40S ribosomal protein S8
SDH	Sb02g002570.1	Sorbitol dehydrogenase homolog1
Serpin-ZXA	Sb01g014740.1	Serpin-ZXA-like
SMP-PM41	Sb03g029840.1	Seed maturation protein PM41
SOD-4A	Sb01g035350.1	Superoxide dismutase-4A
SUS	Sb04g030600.1	Sucrose synthase
SUS2	Sb06g000580.1	Sucrose synthase2
TCP-1	Sb05g022470.1	TCP-1/cpn60 chaperonin family protein
Trx H	Sb02g004850.1	Thioredoxin H-type
v-ESP	Sb01g012640.1	Vicilin-like embryo storage protein

Table S2. Differential expressed proteins of kernels and abundance changes in two maize contrasting hybrid lines to drought between drought treatment and well-watered control.

GI [a]	Protein Name [b]	Coverage (%) [c] /Peptide Fragments [d]	Fold Change (B73) [e]	Fold Change (Lo964) [e]
		Cell rescue/defense		
219363419	Dehydrin	23.0/6	1.64 ± 0.09	−1.02 ± 0.13
295856	RAB-17	37.5/10	1.36 ± 0.09	1.01 ± 0.25
226491145	Late embryogenesis abundant protein Lea14-A	74.3/17	1.46 ± 0.15	1.04 ± 0.14
195659191	Embryonic abundant protein 1	84.6/25	1.47 ± 0.15	−1.48 ± 0.08
226499304	Pathogenesis-related protein 10	11.2/2	1.27 ± 0.08	−1.30 ± 0.03
226531123	Glycine-rich protein 2b	44.2/3	1.61 ± 0.20	1.42 ± 0.09
226492587	Stress-inducible membrane pore protein	63.2/15	1.32 ± 0.07	1.01 ± 0.24
226496775	Xylanase inhibitor protein 1	24.5/3	1.63 ± 0.04	−1.31 ± 0.02
293332305	Serpin-ZXA-like	13.6/5	1.30 ± 0.05	−1.02 ± 0.14
293335211	Heavy-metal-associated domain-containing protein	11.9/2	1.54 ± 0.52	1.00 ± 0.16
473187	Protein kinase C inhibitor	24.2/2	−1.46 ± 0.04	1.06 ± 0.21
75994217	Hageman factor inhibitor	18.7/2	−1.45 ± 0.03	−1.02 ± 0.19
		Redox homeostasis		
257333334	glutathione transferase 30	21.2/8	1.40 ± 0.14	−1.42 ± 0.02
48374955	glutathione peroxidase	44.0/8	1.33 ± 0.08	−1.02 ± 0.16
226494622	Glutathione *S*-transferase,-like protein	19.8/3	1.44 ± 0.06	1.07 ± 0.21
257728955	Grx_C2.2—glutaredoxin subgroup I	69.9/8	1.26 ± 0.10	1.34 ± 0.09
87133468	1-Cys peroxiredoxin antioxidant	76.4/57	1.32 ± 0.08	1.04 ± 0.18
195652835	Thioredoxin H-type	61.5/9	1.33 ± 0.09	1.04 ± 0.16
7548002	Mn-superoxide dismutase	47.6/18	1.59 ± 0.18	1.00 ± 0.20
6018746	Superoxide dismutase-4A	46.7/6	1.28 ± 0.06	1.07 ± 0.17
238008410	NADH-ubiquinone oxidoreductase subunit B17.2	18.2/2	1.53 ± 0.01	1.13 ± 0.10
195655511	NAD dependent epimerase/dehydratase	24.3/2	1.64 ± 0.05	1.35 ± 0.07
		Hormone response		
226497210	ABA-responsive protein	23.5/5	1.46 ± 0.04	1.01 ± 0.19
226498678	Ethylene-responsive protein	28.7/6	1.50 ± 0.19	1.00 ± 0.17
226508662	Ethylene-responsive protein	27.2/7	1.49 ± 0.15	1.01 ± 0.08
301069326	Auxin response factor 23	9.56/1	1.52 ± 0.19	1.35 ± 0.09
224028639	Jasmonate-induced protein	26.8/2	−1.54 ± 0.008	−1.31 ± 0.17
		Signal transduction		
229611800	GTP-binding nuclear protein Ran-A1	40.1/11	−1.35 ± 0.04	−1.20 ± 0.01
226528736	ATP/GTP binding protein	16.1/4	1.34 ± 0.08	1.05 ± 0.18
255037841	Histidine kinase	7.52/2	−1.51 ± 0.02	1.28 ± 0.11
226493048	Nucleoside diphosphate kinase 4	23.5/4	1.35 ± 0.09	1.05 ± 0.20
254256262	Pyruvate orthophosphate dikinase 1	23.0/12	−2.11 ± 0.04	−1.43 ± 0.05

Table S2. *Cont.*

GI [a]	Protein Name [b]	Coverage (%) [c] /Peptide Fragments [d]	Fold Change (B73) [e]	Fold Change (Lo964) [e]
Storage proteins				
228310	Globulin 2	77.6/156	1.07 ± 0.20	−1.51 ± 0.12
224030527	Globulin 1	70.5/205	1.41 ± 0.10	−1.10 ± 0.23
195658011	Globulin-1 *S* allele precursor	77.6/152	1.63 ± 0.22	1.06 ± 0.02
226500532	Seed maturation protein PM41	18.0/9	1.42 ± 0.08	1.09 ± 0.25
22284	Vicilin-like embryo storage protein	69.8/180	−1.04 ± 0.33	1.46 ± 0.001
330732090	γ-zein	2.27/1	−1.67 ± 0.01	−1.13 ± 0.006
Protein biosynthesis				
9931636	Ribosomal protein s6 RPS6-2	23.1/5	1.65 ± 0.26	−1.04 ± 0.29
257667240	40S ribosomal protein S18	54.6/7	1.52 ± 0.09	1.07 ± 0.13
6226702	40S ribosomal protein S8	33.5/7	1.81 ± 0.10	−1.01 ± 0.23
226502084	60S ribosomal protein L7a	19.8/5	1.50 ± 0.14	−1.05 ± 0.10
258598734	QM-like protein	23.7/4	1.36 ± 0.08	−1.01 ± 0.13
Protein folding and assembly				
7546186	Heat shock protein HSP82	23.1/9	−1.65 ± 0.03	1.14 ± 0.08
293331695	HSP protein	23.3/10	−1.04 ± 0.21	−1.51 ± 0.04
54299342	Mitochondrial small heat shock protein 22	44.0/7	−1.01 ± 0.09	−1.39 ± 0.02
293335765	TCP-1/cpn60 chaperonin family protein	8.94/2	1.02 ± 0.13	−1.34 ± 0.09
453670	Heat shock protein 26	57.5/9	1.00 ± 0.09	−1.44 ± 0.07
257745378	Peptidyl-prolyl cis-trans isomerase Family protein	19.5/3	1.08 ± 0.23	1.29 ± 0.02
226495869	Histone deacetylase 6	6.49/2	1.45 ± 0.13	–1.91 ± 0.09
59861271	Protein disulfide isomerase	6.56/2	1.90 ± 0.29	1.08 ± 0.23
Protein degradation				
224029787	Proteasome subunit alpha type	49.2/8	1.40 ± 0.12	−1.26 ± 0.04
226531007	Proteasome subunit alpha type 1	22.3/7	1.32 ± 0.08	−1.17 ± 0.02
Carbohydrate metabolism				
22488	Sucrose synthase	22.8/10	−1.73 ± 0.03	−1.42 ± 0.06
3342802	Cytosolic 6-phosphogluconate dehydrogenase	23.6/7	1.21 ± 0.04	−1.39 ± 0.003
226530488	Glucose-6-phosphate 1-epimerase-like	11.9/3	1.28 ± 0.05	1.02 ± 0.26
194688844	Sucrose synthase2	29.5/11	1.61 ± 0.15	−1.01 ± 0.07
293333951	Isocitrate dehydrogenase [NAD] Regulatory subunit 1	20.9/4	−1.10 ± 0.23	−1.41 ± 0.14
226504732	Sorbitol dehydrogenase homolog1	56.2/27	2.08 ± 0.10	1.39 ± 0.05
226499336	Succinate dehydrogenase flavoprotein subunit	12.9/2	1.45 ± 0.14	1.01 ± 0.24
291047790	Succinate semialdehyde dehydrogenase	23.1/8	1.41 ± 0.12	1.10 ± 0.04
257726331	Cytokinin-*O*-glucosyltransferase 2	7.54/2	1.40 ± 0.04	1.08 ± 0.25
Transcription factor				
226496988	Fibrillarin-2	20.5/2	1.51 ± 0.13	−1.29 ± 0.15
308081068	AP2-EREBP-type transcription factor	12.9/2	1.33 ± 0.06	−1.04 ± 0.34

Table S2. *Cont.*

GI [a]	Protein Name [b]	Coverage (%) [c] /Peptide Fragments [d]	Fold Change (B73) [e]	Fold Change (Lo964) [e]
Amino acid and lipid metabolism				
227478191	Argininosuccinate synthase	12.3/3	1.78 ± 0.23	1.27 ± 0.08
293332891	Isovaleryl-CoA dehydrogenase	13.0/2	1.77 ± 0.14	1.00 ± 0.31
195605626	Oleosin 16 kDa	41.0/14	1.32 ± 0.07	−1.30 ± 0.003
Secondary metabolism				
226500722	Anthocyanidin 5,3-*O*-glucosyltransferase	5.86/2	1.23 ± 0.02	−1.33 ± 0.11
293335591	Dihydrolipoyl dehydrogenase	15.0/6	1.32 ± 0.11	−1.06 ± 0.25
52699545	Isopentenyl-diphosphate delta isomerase 2	8.72/2	1.47 ± 0.15	−1.01 ± 0.06
596080	Thiamine biosynthetic enzyme	16.4/4	1.39 ± 0.12	1.57 ± 0.23
Membrane and transport				
75278333	Aquaporin TIP1-1	6.4/3	1.34 ± 0.09	−1.08 ± 0.31
75308033	Aquaporin TIP3-2	7.86/2	1.41 ± 0.05	1.84 ± 0.05
293332063	Cytochrome b5	11.9/2	–1.41 ± 0.11	1.25 ± 0.51
Others and unknown				
226495887	Adaptin ear-binding coat-associated protein 1	12.8/1	1.25 ± 0.04	−1.33 ± 0.17
219363567	Hypothetical protein LOC100217226	9.47/1	–1.71 ± 0.10	−1.35 ± 0.06
219363597	Hypothetical protein LOC100216972	12.0/1	1.32 ± 0.13	−1.77 ± 0.17
226528357	Hypothetical protein LOC100278634	34.8/3	1.59 ± 0.18	1.08 ± 0.20

[a] GI number in National Center for Biotechnology Information (NCBI) genebank database; [b] The identified proteins were named according to the annotations in NCBI database. According to the comments in NCBI, many of these proteins were annotated based on homology evidence (Arabidopsis, rice, *etc.*). A protein with identify or significant homology to a known protein was annotated as the same name. A protein without any functional annotations was annotated as "hypothetical" protein; [c] Sequence coverage (%) is calculated by dividing the number of amino acids in the peptide fragments observed by the protein amino acid length; [d] Peptide fragments were referred to the number of matched peptide fragments generated by trypsin digestion; [e] Fold change is expressed as the ration of intensities of up-regulated (positive value) or down-regulated (negative value) proteins between drought stress treatments and control (well-water condition). Fold change SD is presented as mean standard deviation (SD).

Table S3. The results of protein level FDR analyses from two biological replicates. The FDR analysis was performed using the Proteomics System Performance Evaluation Pipeline integrated with the ProteinPilot.

Number of Proteins Detected			
**Critical FDR**	**Local FDR**	**Global FDR**	**Global FDR from Fit**
Biological replicate 1			
1.0%	648	705	706
5.0%	671	763	762
10.0%	681	824	827
Biological replicate 2			
1.0%	624	670	668
5.0%	633	719	732
10.0%	638	809	807

Figure S1. A heat map of protein expression (red, high expression; green, low expression) in response to drought stress in kernels of the tolerant maize genotype Lo964 and the sensitive maize genotype B73. Group-I refers to the abundances of proteins increased in both two genotypes under drought stress conditions, but relatively lower increase degree in maize drought-sensitive line B73; the abundances of the group-II proteins increased in B73 and no obvious changes in Lo964, but relatively higher increase degree in drought-sensitive line maize B73; proteins of group-III showed decreased trends in Lo964 and a high decrease degree in Lo964 than in B73; those of group-IV had an increase profile in Lo964 but decrease one in B73, the proteins of this group showed a much great degree changes between two genotypes.

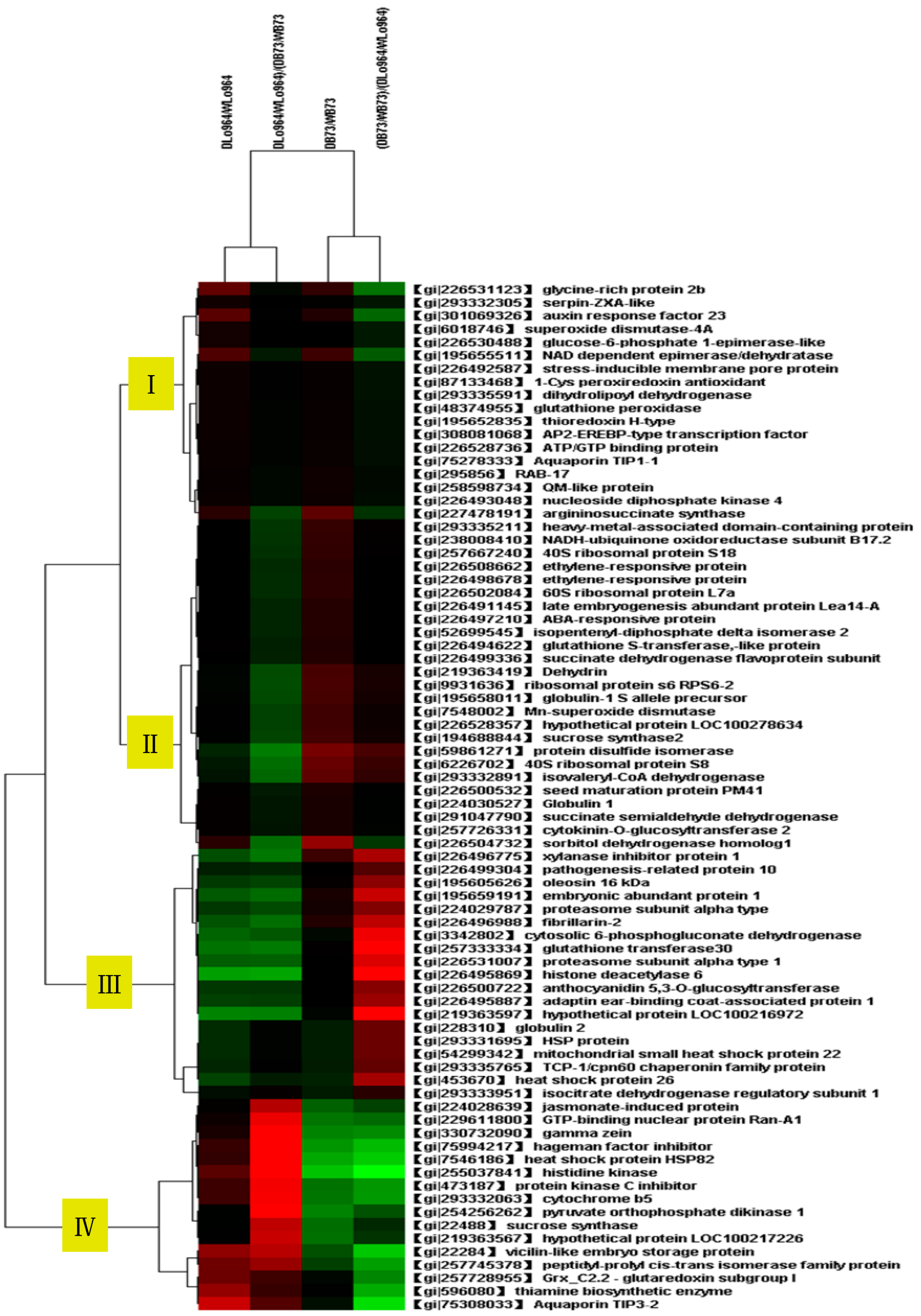

Figure S2. Gene Ontology (GO) analyses of drought-responsive proteins in the kernels of Lo964 (**A**,**C**,**E**) and B73 (**B**,**D**,**F**). All identified proteins were included and presented in biological process (**A**,**B**), molecular function (**C**,**D**) and cellular component (**E**,**F**). The red circles in **C** and **D** represent the re-categorization of "binding" group.

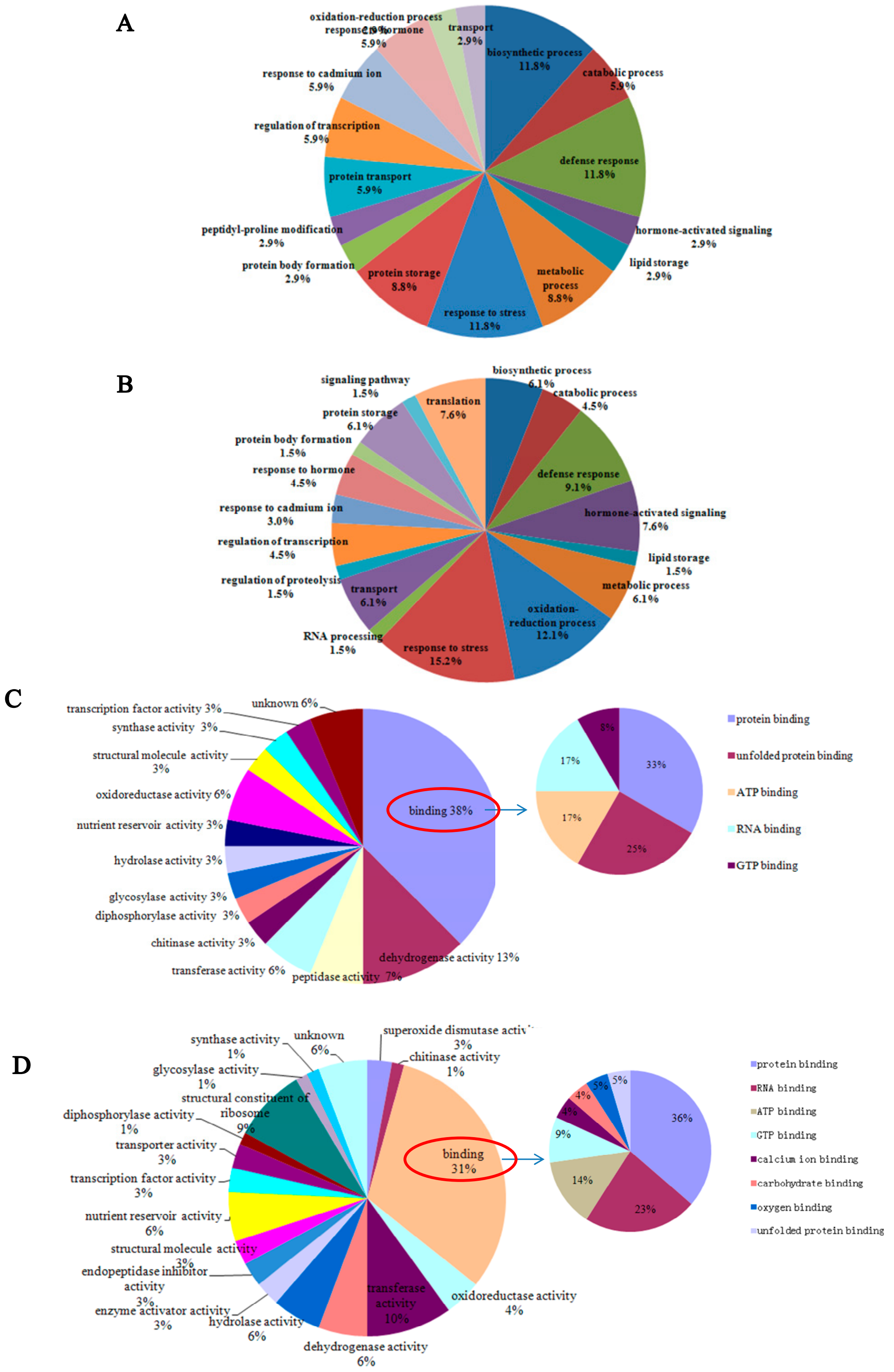

Figure S2. *Cont.*

E

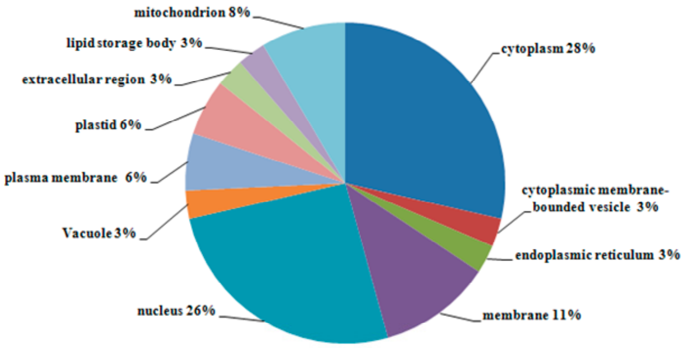

F

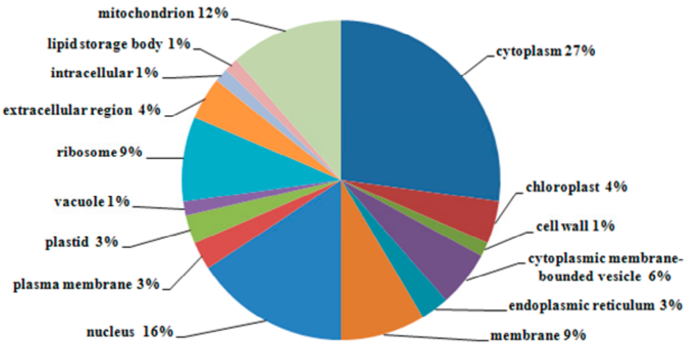

Figure S3. ROS generation and scavenging pathways associated with proteins in maize kernels that were up- or down regulated under drought stress treatments. Superoxide was generated by NADH-ubiquinone oxidoreductase (UOR), and further dismutated to hydrogen peroxide (H_2O_2) by superoxide dismutase (SOD). H_2O_2 can be scavenged by glutathione peroxidase (GPX) and reduced 1-Cys peroxiredoxin antioxidant (1-Cys Prx) using thioredoxin H-type (Trx-H) as electron donors. H_2O_2 and reduced monomeric glutathione were also catalyzed by GPX to decrease H_2O_2 level. Glutathione *S*-transferase (GST) and glutathione transferase30 (GT30) catalyze the conjugation of the reduced form of glutathione (GSH) to xenobiotic substrates for the purpose of detoxification. During this process, glutaredoxin (Grx), as a redox enzyme, was reduced by the oxidation of glutathione. Red and green arrows indicated the up-regulated or down-regulated trends of proteins under drought stress in maize genotypes B73 and Lo964, respectively.

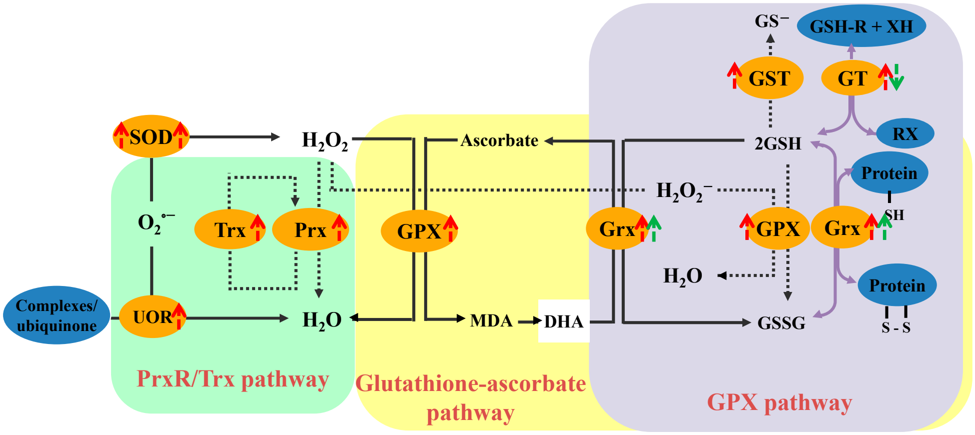

Figure S4. Transcription and protein metabolic processes associated with proteins in response to drought stress in kernels of the sensitive maize genotype B73 and the tolerant maize genotype Lo964. Blue and green arrows indicated the up-regulated or down-regulated trends of proteins under drought stress in maize genotypes B73 and Lo964, respectively. The red arrows represent the flow direction of genetic information.

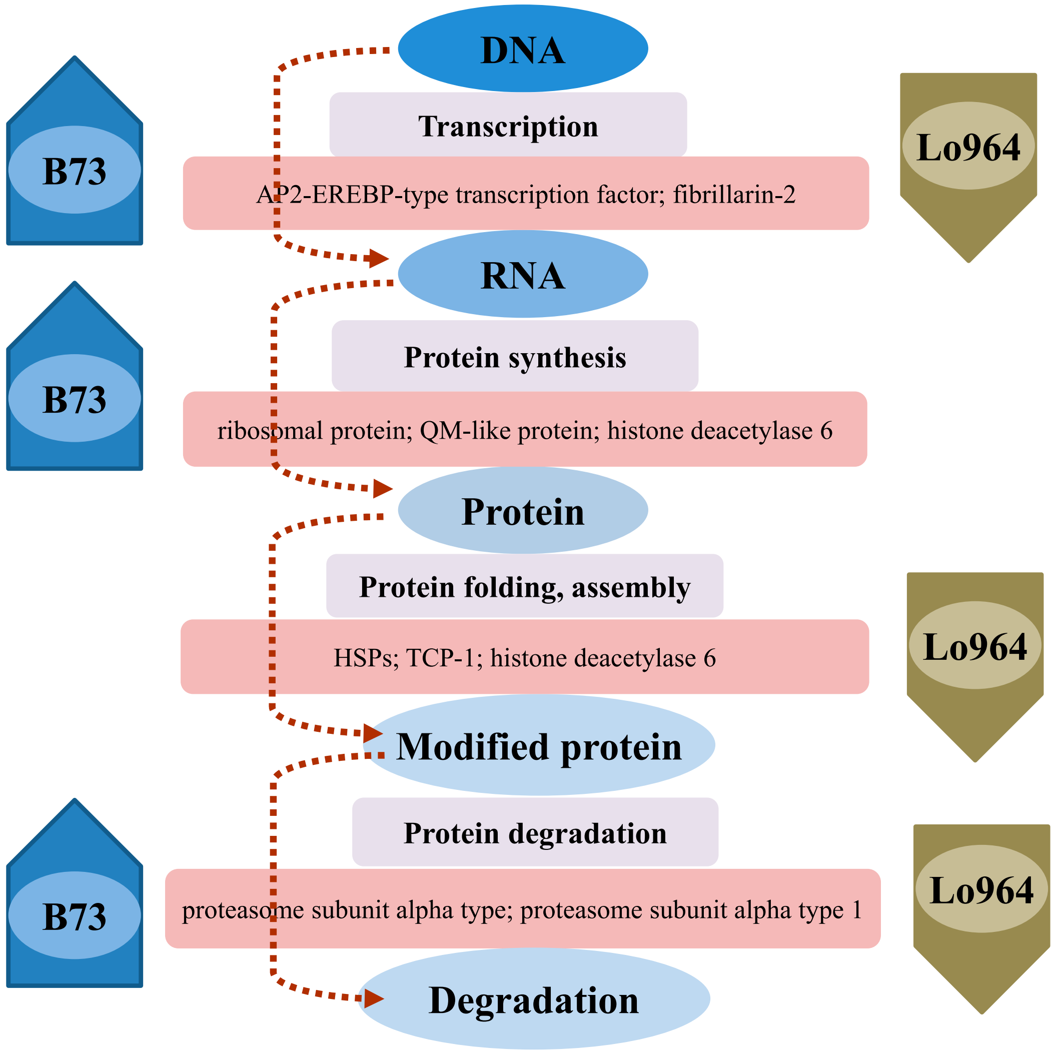

Supplement: Supplementary File 1 [file ijms-15-18892-s001.pdf]
